# Evaluation of potential immunogenicity differences between *Pandemrix*™ and *Arepanrix*™

**DOI:** 10.1080/21645515.2016.1168954

**Published:** 2016-04-22

**Authors:** Quentin Canelle, Walthère Dewé, Bruce L. Innis, Robbert van der Most

**Affiliations:** GSK Vaccines, Rixensart, Belgium

**Keywords:** antibody, AS03, Arepanrix, avidity, H1N1, hemagglutinin, immunogenicity, narcolepsy, Pandemrix, vaccine

## Abstract

In retrospective observational studies, an increased relative risk of incident narcolepsy was observed in some European countries among recipients of the AS03-adjuvanted, A(H1N1)pdm09, inactivated, detergent-split virion vaccine *Pandemrix*™ manufactured in Dresden, Germany (D-Pan H1N1). A similar increased risk was not observed in a retrospective population-based study in individuals in Quebec province, Canada, who received *Aprepanrix*™, a Quebec-manufactured AS03-adjuvanted A(H1N1)pdm09 inactivated, detergent-split virion vaccine (Q-Pan H1N1). Antibody responses in D-Pan *versus* Q-Pan vaccinees (adults/children) measured as hemagglutination inhibition (HI) titers 21 d post-vaccination were found to be equivalent (NCT01161160). The current post-hoc analysis was conducted to determine whether antibody avidity differed following immunization with the 2 vaccines. Using surface plasmon resonance, we evaluated the capacity of serum specimens (drawn from the comparative immunogenicity trial) from a subset of subjects aged 3–9 y who received either D-Pan or Q-Pan (N = 28/group), to bind to recombinant A(H1N1)pdm09 hemagglutinin. IgG antibodies were purified from Day 21 sera. Binding was assessed by end association level; dissociation by retention of antigen-antibody complexes at the end of the dissociation phase, and k_d_. Inter-run variability for the control monoclonal antibody, association levels and dissociation levels was low (CVs 1.3%, 7.8% and 1.4%, respectively); non-specific binding was negligible. High avidity and slow dissociation was observed for both groups (k_d_ ≤ 10^−4^/s; geometric mean [IQR] association and dissociation levels for D-Pan/Q-Pan: 15.4 RU [13.4–17.7]/12.4 RU [10.8–14.3] and 94.5% [92.5–96.5]/95.5% [93.5–97.6], respectively). Association, but not dissociation levels correlated with HI titers. No significant differences in avidity parameters were observed between D-Pan and Q-Pan sera.

## Introduction

After the declaration of the H1N1 influenza pandemic in 2009, H1N1 vaccines were widely administered. In certain countries in Europe, vaccination campaigns firstly targeted the most susceptible populations, including children, who had priority access to the vaccine. However, the vaccination coverage of children was very heterogeneous across European countries (range 0.2–74%).^1^ Of the approximately 40 million vaccinees in the European Union (EU), over 30 million received *Pandemrix*™ (D-Pan H1N1), a pandemic H1N1 influenza vaccine manufactured by GSK in Dresden, Germany, which contained the inactivated pandemic A/California/7/2009 (A(H1N1)pdm09) strain adjuvanted with AS03.^2,3^ The vaccine was indicated for the prophylaxis of influenza in an officially declared pandemic situation, and, since 2010 post pandemic, for the prophylaxis of influenza caused by the A(H1N1)pdm09 virus in the EU, if trivalent seasonal vaccines were unavailable. Since the end of the 2010–11 northern hemisphere influenza season, the vaccine was no longer being used or produced.

An increased incidence of narcolepsy after D-Pan vaccination has been reported, particularly among children and adolescents, with the first reports emerging in August 2010.^4^ Narcolepsy is a rare chronic disorder characterized by excessive day-time sleepiness and episodes of cataplexy due to the loss of hypocretin-producing neurons, and likely has an autoimmune basis.^5^ In 2012, GSK submitted a research plan to the European Medicines Agency aiming to further investigate the potential association between D-Pan vaccination and the narcolepsy signal.^6^ Since the relative risk of narcolepsy has not been observed as similarly increased for vaccinees in the Canadian Quebec province who received *Aprepanrix*™ (Q-Pan H1N1),^7^ an AS03-adjuvanted A(H1N1)pdm09 vaccine manufactured in Quebec City, one research objective was to evaluate the potential immunogenicity differences between D-Pan and Q-Pan in terms of the quality of the antibody responses they elicited following immunization. The vaccines contained the same quantities of split-virion antigen (measured as hemagglutinin [HA] concentration) and the same dose of AS03. The two manufacturing sites where the detergent-split virion vaccines were prepared have used similar processes, however there were some differences which mainly occurred during downstream purification of the antigens.^8^ Recently, several differences between the D-Pan and Q-Pan vaccine antigens were reported, including variations in one HA amino acid residue (146N) and in the amount of viral nucleoprotein (NP).^9-11^ Yet, based on the hemagglutination inhibition (HI) titers induced by D-Pan or Q-Pan at 3 weeks post vaccination (Day 21) in both adults and children, the vaccines were found to be immunogenically equivalent (*i.e.*, having a 95% confidence interval of the geometric mean titer [GMT] ratio between 0.5 and 2).^12^ Notably, immunogenic equivalence with respect to HI antibody titers had also been demonstrated for AS03-adjuvanted H5N1 vaccines manufactured at the Dresden and Quebec sites.^13^

Antibody avidity reflects the accumulated binding strength of multiple antigen-antibody interactions.^14^ The relevance of the qualitative aspects of the humoral response, such as antibody avidity, for protective immunity has been highlighted for a variety of pathogens, e.g. ref.^15-18^, although this has been contradicted by some reports.^19-21^ During the A(H1N1)pdm09 pandemic, high-avidity antibodies have been linked to increased virus neutralization and less severe symptoms of infection,^22^ while high-titered, high-avidity non-neutralizing antibody responses after influenza vaccination have been associated with severe influenza.^23^ Avidity is generally determined by chaotropic ELISA, and occasionally by surface plasmon resonance (SPR)-based spectroscopy (e.g., in refs.^24-26^). The latter method is considered by some authors as more accurate and relevant,^27-29^ because the near-physiological conditions under which it is performed preclude alteration of the antigen-antibody complex, and consequently the measured avidity provides a more correct reflection of the actual binding strength and kinetics. In SPR, biosensors measure the mass perturbations resulting from the analyte binding to the (antigen) ligand immobilized on the biosensor chip. The high sensitivity of SPR has been illustrated by its ability to detect differences in antibody avidity between elderly and young adult A(H1N1)pdm09 vaccinees.^25^

The current post-hoc evaluation was performed with the objective to investigate whether IgG antibodies elicited by the D-Pan and Q-Pan vaccines in children differ in terms of avidity. Using SPR, we assessed the serum samples obtained at Day 21 from children aged 3–9 years, taken from the above-mentioned primary clinical study in which HI responses to the 2 vaccines were compared.^12^ As part of this analysis, we determined whether kinetic binding parameters (the end association [binding] rate and the dissociation rate) correlated with the HI titers. The evaluation was undertaken to address the question whether potential specific antigenic differences in the HA protein between the 2 vaccines may have contributed to the divergent observed associations with narcolepsy for D-Pan in some European countries and for Q-Pan in Canada.

## Results

SPR-based avidity testing of the serum samples of the D-Pan and Q-Pan groups (in duplicate) was performed based on a described technology.^24-26^ Purified recombinant A(H1N1)pdm09 full-length HA (rHA0) was used as the immobilized ligand. Prior to quantitatively comparing the group avidity parameters, we optimized the SPR analysis method using 28 samples per vaccine group. Based on the outcomes, 1 or 2 samples per group were discarded (Table S1), and the remaining samples were used in the statistical analyses.

### SPR analysis optimization

#### Sample preparation by purification of the IgG fraction

The IgG fraction was purified with the aim to reduce non-specific binding of the serum matrix and to remove IgM, since differences in the IgM fraction could confound the IgG measurements. During the purification process, the IgG-containing fraction is retained, whereas the other serum proteins are discarded. Since this process also results in sample dilution, we compared the HI antibody titers of the unpurified samples with those of the purified, IgG-containing sample fractions.

All obtained HI titers exceeded 1:40 before purification and had decreased by approximately 50% after purification, but all remained at or above 1:40 (Table S1). For both the Q-Pan and D-Pan group, there was a positive correlation between post- and pre-purification HI titers (r = 0.83 and r = 0.77, respectively). This suggests that the impact of purification on HI titers was mainly attributable to dilution, although a minor contribution from other factors, such as the removal of IgM, cannot be excluded.

#### HA-specific binding level determination

Using the purified IgG-containing fractions, we determined the level of non-specific (background) binding of the serum matrix that remained present after the purification step, as measured on the biosensor's reference channel. We also assessed the stability of the biosensor chip's binding capacity, and established the rate of HA-antibody binding that was reached at the stability point, at 600 s (the ‘end association rate'), for the control (an A(H1N1)pdm09 HA-specific mouse monoclonal antibody [mAb]).

For the majority of samples, non-specific binding was close to baseline (Fig. S1). Only one sample (of the D-Pan group) showed very high non-specific binding (∼70 resonance units [RU]) and was therefore not used for further statistical analyses.

The low statistical variation in the end association rates for 10 injections of the control mAb (coefficient of variation [CV] 1.3%; mean 29.7 RU) suggested that the binding capacity of the biosensor chip was conserved throughout the experiment (data not shown).

### Avidity assessments by SPR

We next evaluated the capacity of the purified D-Pan and Q-Pan serum samples to bind to rHA0. The avidity parameters evaluated were the end association rate, the dissociation rate (the fraction of HA-antibody complexes that remained bound at the end of the dissociation phase [1000 s]) and the dissociation rate constant k_d_. The k_d_ values were determined from the data obtained during the dissociation phase by the use of a 1:1 model, which corresponded to the fitting of the equation $R=R_{0}*e^{-(k_{d}(t-t_{0}))}$(where R/R_0_: binding responses at time-points t/t_0_, respectively; t_0_: stability report point, and t: the end of the 1000 s dissociation period). HA-specific signals were corrected for background binding on the reference channel and for the signal of the blank. The distributions of avidity parameter values and HI titers were characterized after a log_10_ transformation.

#### Association and dissociation parameters

**Figure 1.**
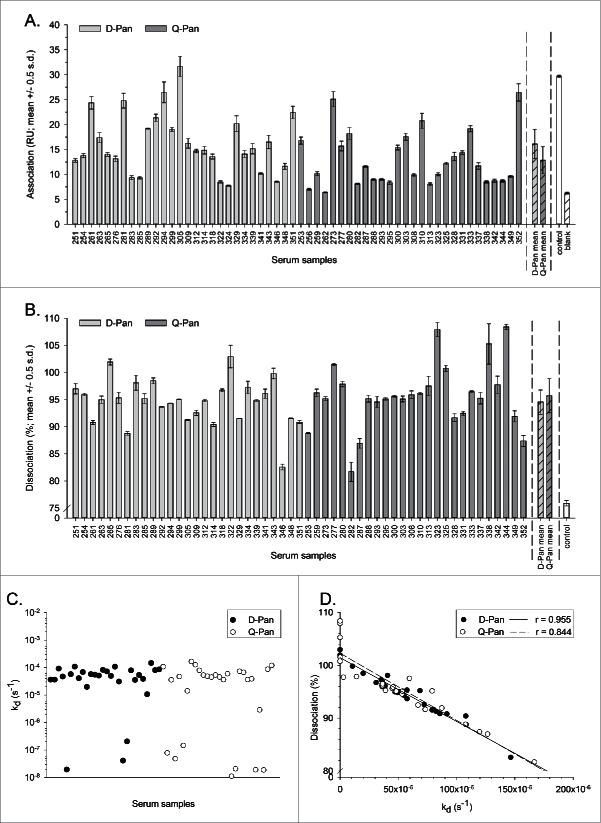
Avidity parameters per vaccine group. The end association rate (binding response at the end of the association phase; A), dissociation rate (fraction of complex that remained bound at the end of the dissociation phase; B), dissociation rate constant k_d_ (C), and correlation between the dissociation rate and the k_d_ (D) were assessed for serum samples obtained 3 weeks after vaccination from children who received either the D-Pan or Q-Pan vaccine. Bars and error bars represent the means of 2 analysis runs per sample and the standard deviation (s.d.), respectively. HA-specific signals were corrected for background binding on the reference channel and for the signal of the blank. Control, an A(H1N1)pdm09 HA-specific mouse monoclonal antibody. RU, resonance units.

For both groups, the end association and dissociation rates and the k_d_ values were indicative of high avidity, i.e., strong binding and relatively slow dissociation kinetics (Table 1**;** Fig. 1). Importantly, the 2 dissociation parameters assessed (dissociation rate and k_d_) correlated well for both the D-Pan and the Q-Pan group (r = 0.95 and 0.84 respectively), suggesting that both parameters were relevant and described the dissociation kinetics in a similar manner.

**Table 1.** HI titers and avidity parameters.

		Geometric Mean (95% CI)		GMR _Q-Pan/D-pan_
	Data range across groups^@^	D-Pan	Q-Pan	(95% or 97.5%CI^#^)
HI titers pre-purification	80–3620	929 (677–1273)	814 (590–1122)	0.88 (0.56–1.37)
HI titers purified IgG fraction	57–1280	453 (338–607)	413 (306–556)	0.91 (0.60–1.39)
Association rates (RU)^*^	7.7–34.5	15.4 (13.4–17.7)	12.4 (10.8–14.3)	0.81 (0.64–1.01)
Dissociation rates (%)^*^	79.2–110.5	94.5 (92.5–96.5)	95.5 (93.5–97.6)	1.01 (0.98–1.05)
k_d_ (s^−1^)^†^	1.1 × 10^−8^–1.7 × 10^−4^	—	—	—

@ Data shown are based on the individual analysis runs, excluding discarded samples.

# Geometric mean ratios (GMR) were estimated with 95% confidence intervals (CI) for HI titers, and with 97.5% CI for association or dissociation rates (to account for multiplicity).

* Geometric means of association and dissociation rates determined during 2 analysis runs were computed by sample.

† No statistical analyses were performed for the k_d_ due to the presence of outliers in the log_10_-transformed data. RU, resonance units.

**Figure 2.**
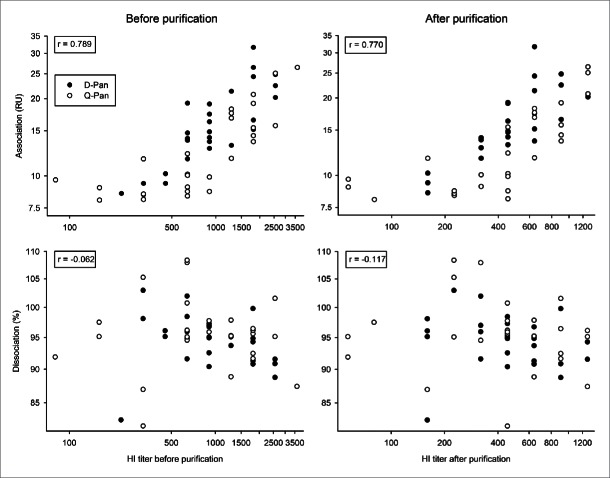
Relationship between HI titers and avidity parameters before and after sample purification. Serum samples were obtained 3 weeks after vaccination from children who received either the D-Pan or Q-Pan vaccine. Samples were purified to reduce the fractions of IgM and non-specific protein. End association and dissociation rates were assessed on the serum samples before purification, as well as on the purified IgG fraction (left-hand and right-hand panels, respectively). Symbols represent the averages of 2 analysis runs per sample. The r correlation (indicated in the left-hand corner of each graph) was determined irrespective of the vaccine group. RU, resonance units. HI titer, hemagglutination inhibition titer.

The correlation between HI titers and the association or dissociation rate was assessed irrespective of the vaccine group. In contrast to the dissociation rate, which is only k_d_-dependent, association rates are dependent on the specific antibody concentration and affinity (k_d_/k_a_; where k_a_ is the association rate constant). Perhaps consistently, for both the pre- and post-purification HI titers, we found no correlation with the dissociation rate and a positive correlation with the end association rate (Fig. 2).

Overall, the SPR method yielded analytically acceptable results. The end association rates were highly reproducible (with an average CV of 7.8%), and only 2 samples (both of the Q-Pan group) yielded end association rates that were considered unsuitable to include in further statistical analyses (*i.e.*, differing less than 1 RU with the signal of the blank). Dissociation rates for the control mAb (mean 75.9%, CV 1.4%) were in all cases lower than those measured for the serum samples. For some samples, dissociation levels exceeded 100%, which corresponded with k_d_ values at or below 10^−7^ s^−1^. These values should be interpreted with caution, since this apparent increase in binding during the dissociation phase may be the result of the correction applied for the background binding on the reference surface, with the samples in question behaving differently on the reference surface as compared to the HA surface.

#### Comparison of avidity parameters between groups

**Figure 3.**
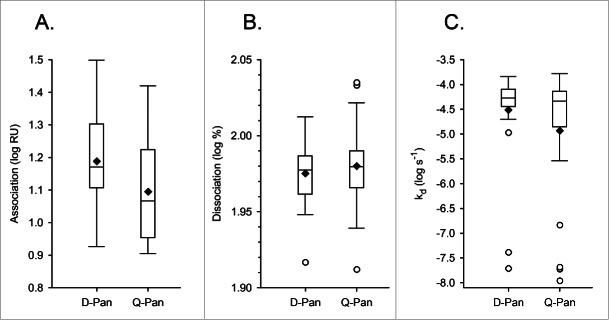
Distribution of avidity parameters. Distributions of the log_10_-transformed data for the end association rate (A), dissociation rate (B) and the dissociation rate constant k_d_(C) were determined using serum samples obtained 3 weeks after vaccination from children who received either the D-Pan or Q-Pan vaccine. Box-and-whiskers plots represent the medians and interquartile ranges (boxes), and the minimum and maximum values (whiskers). Diamonds and open circles represent means and outliers, respectively. Values above the 75^th^ percentile + 1.5 IQR, or below the 25^th^ percentile – 1.5 IQR, were considered outliers. RU, resonance units.

The main research objective was to compare the kinetic parameters of antibody binding to rHA0 between the D-Pan and Q-Pan groups. There was no evidence of a significant difference between groups for either the end association or dissociation rate (Table 1; Fig. 3AB). Yet, a difference in the end association rates could not be excluded, as the upper limit of the geometric mean ratio (GMR) confidence interval was found to be close to 1.

**Figure 4.**
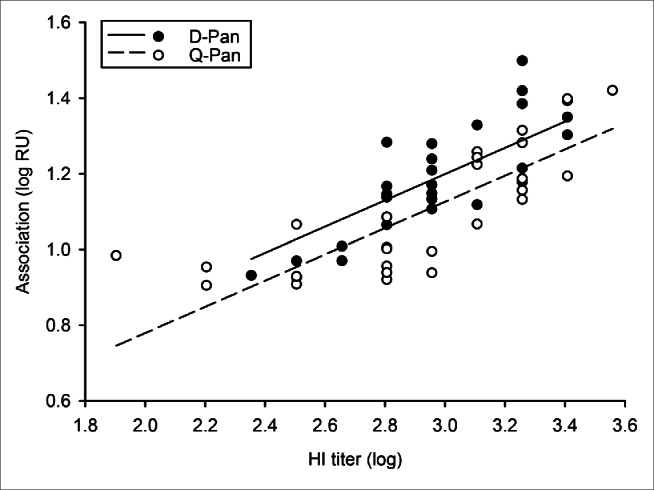
Relationship between end association rates and HI titers after adjustment for the antibody concentration. The correlation between the log_10_-transformed data of the end association rate and hemagglutination inhibition (HI) titers was assessed after adjustment for the antibody concentration. Serum samples were obtained 3 weeks after vaccination from children who received either the D-Pan or Q-Pan vaccine. Symbols represent the averages of 2 analysis runs per sample. Regression lines were estimated by analysis of covariance modeling. RU, resonance units.

Given the observed correlation between post-purification HI titers and end association rates, and because there was a minor difference in post-purification HI titers between groups (Fig. S2; Table 1), we next evaluated the potential difference in end association rates after adjustment for the antibody concentration (Fig. 4; Table 2). Based on the performed supplementary analysis of covariance, we found no evidence that the observed trend in the association rates could be explained by a difference in the HI titers.

**Table 2.** Geometric mean (ratio) of the association rate after adjustment for the antibody concentration.

	Geometric mean (95% CI) of the association rate, in RU		
Covariate	D-Pan	Q-Pan	GMR _D-Pan/Q-Pan_ (95% CI) of the association rate, in RU
log_10_HI titer (pre-purification)	15.1 (13.8–16.4)	12.7 (11.7–13.9)	0.84 (0.75–0.96)
log_10_ HI titer (purified IgG fraction)	15.2 (13.9–16.6)	12.7 (11.6–13.9)	0.83 (0.73–0.95)

GMR, geometric mean ratio.

Due to the presence of outliers in the log_10_-transformed k_d_ values (Fig. 3C), no further statistical analyses were performed for this parameter.

## Discussion

Although case reports of narcolepsy after D-Pan vaccination have emerged in several European countries, a similar observation has not been made in a retrospective population-based study in Q-Pan vaccinees in Quebec.^7^ Consistently, a recent report of post-marketing safety surveillance data in Ontario, Canada did not reveal any possible narcolepsy cases among Q-pan vaccinees.^30^ The results of the Quebec study prompted the question whether vaccine-induced antibody responses vary between the 2 vaccines. Since the immunogenic equivalence of D-Pan and Q-Pan vaccines in terms of HI titers has been demonstrated in the primary study,^12^ we used in the current *post-hoc* evaluation a subset of these samples to investigate whether differences in antibody avidity exist between the responses induced by the 2 vaccines. We performed an SPR-based avidity analysis to evaluate the capacity of the D-Pan and Q-Pan sera to bind to rHA0, by assessing the end association and dissociation rates and the k_d_.

Our results showed no evidence of a significant difference in the end association and dissociation rates between the vaccines, which is consistent with the primary study results demonstrating equivalence of HI titers.^12^ We do not expect that the 3 single-residue differences between the sequences of the vaccines and/or the ligand would have affected the results, because the kinetic parameters measured for the polyclonal serum samples represented the sum of the values obtained at the individual antibody level, which were likely to complement each other. Moreover, even if such variation would result in a detectable impact on the avidity parameters of a sample, this impact would likely be similar for both vaccines, since the same ligand was used for all the analyses. On a separate note, our data showing that the dissociation rate did not correlate with the HI titers may be in line with the fact that dissociation (in contrast to association) is theoretically concentration-independent, although the exact concentrations of the different antibody populations in the polyclonal sera are unknown.

The high-avidity antibodies detected for both vaccines reflected a slow decay of the rHA0 antigen-antibody complexes. Though we did not include a non-adjuvanted vaccine control group, our results align with the high avidity of the HA-specific antibodies induced by pandemic H5N1 or A(H1N1)pdm09 vaccines containing another oil-in-water based adjuvant (MF59).^24,26^ In these studies, antibody avidity was found to be enhanced in sera from recipients of the MF59-adjuvanted vaccines relative to sera from recipients of non-adjuvanted and/or alum-adjuvanted vaccines.

Overall, the analytical performance of the SPR assay was satisfactory. First, purification of the IgG fraction removed the IgM antibodies (which have higher molecular weights than IgG and may thus interfere with the IgG measurements) and largely reduced non-specific binding, thus enhancing the specificity of the detected signal. Given the naturally lower levels of IgM *vs* IgG in the current age group,^31^ IgM antibodies were in any case expected to account for only a minor fraction of the elicited anti-HA response. Indeed, we previously observed that IgM removal from post-vaccination serum samples from children had no impact on the HI titers of these samples (unpublished data). Second, the high reproducibility of the data obtained with the control mAb between test runs indicated that the integrity and folding of the immobilized HA was maintained (although these results will need to be extrapolated to the polyclonal antibodies in the sera). In addition, the relatively long (600-s) period allowed for sample injection over the rHA0 surface may have contributed to the high data reproducibility, because it ensured that the plateau state of the antibody-antigen interaction was approached. This is important for a correct determination of the interaction's kinetics and strength. Last, it has been suggested that for a reliable k_d_ definition, the decrease in response signals during the dissociation phase should be at least 5%.^32^ Although the dissociation levels in our study indicated that approximately 95% of complex remained bound at the end of the dissociation phase, we found a reassuringly high level of correlation between the dissociation rate and the k_d_, supporting the validity of the k_d_ fitting.

A limited number of studies have used Biacore biosensors to determine antibody avidity in polyclonal sera (e.g. refs.^19,33,34^), and the procedures followed in the current evaluation may contribute to further development of this methodology. Specifically, using the purified IgG fraction as test item and applying a longer sample injection period, as well as basing the data analysis on the dissociation rate rather than on the k_d_ (which may give a closer representation of the biological binding behavior) may have contributed to improve the data quality.

A possible study limitation is that the HA dose of the pediatric D-Pan vaccines used in the primary study, and the dose used in the European countries in which the narcolepsy signal was detected, were different (i.e., 0.9 *vs* 1.9 µg HA, respectively^12,35^). The potential impact of this on the present results is unknown. Nevertheless, for AS03-adjuvanted H5N1 pandemic influenza vaccines it has been observed that a 2-fold change in the antigen dose did not appear to condition the HI antibody response.^36^ Moreover, the use of serum samples from a lower-dose vaccine might have increased the sensitivity to detect immunogenicity variances between treatment groups. As a potential second study limitation, we observed that a borderline difference between the vaccine groups may be present for the end association level, which could not be explained by a difference in HI titers. The sample size of the current study may not have been sufficiently large to detect this difference as statistically significant.

The initial incentive for comparing antibody avidity parameters between the Q-Pan and D-Pan vaccines was the negligible increased risk of narcolepsy observed in Quebec.^7^ The data from the present avidity analysis do not support the hypothesis that the differential narcolepsy risks observed in studies in Europe and Canada are associated with differential avidities of the IgG antibodies. Apparently, the recently reported minor difference between the Q-Pan and D-Pan antigens with respect to the HA amino acid sequences^9^ do not translate into differences in anti-HA antibody avidity. Since the current results thus do not seem to support further investigation into possible variances in antibody avidity, other areas of immunological research might potentially contribute to our understanding of the observed variances in the narcolepsy risks.^10,11^

## Conclusion

The D-Pan and Q-Pan vaccines both elicited high-avidity HA-specific antibodies. The combined HI and avidity data support the conclusion that there are no major immunogenicity differences between the D-Pan and Q-Pan vaccines in terms of the quality of the humoral (IgG) responses to the HA protein. However, these data cannot serve to exclude the existence of minor antigenic differences in the HA proteins between the 2 vaccines.

## Materials and methods

### Serum samples

The 56 serum samples (28 per vaccine group) used in the current study were obtained from a Phase II randomized, controlled study (NCT01161160) conducted in the Philippines and Thailand between January 2010 and January 2011, in which healthy children aged 3 to < 10 years received a single dose of D-Pan or Q-Pan vaccine.^12^ Samples were collected 21 days after vaccination (Day 21). One dose contained a specified volume of an antigen formulation with a HA concentration of 15 μg/ml mixed with AS03_B_, to contain 0.9 μg HA per dose for both the D-Pan and Q-Pan groups. AS03_B_ (elsewhere in this article referred to as AS03) is an Adjuvant System containing α-tocopherol and squalene in an oil-in-water emulsion (5.93 mg tocopherol). The study was approved by local ethic committees and conducted according to good clinical practice and in accordance with the Somerset West 1996 version of the Declaration of Helsinki. Written informed consent was obtained from the parents/guardians of the children before the study procedures.

### Study vaccines

The D-Pan and Q-Pan study vaccines are monovalent, inactivated, detergent split-virion, influenza A(H1N1)pdm09 vaccines (reassortant X-179A strain derived from the A/California/7/2009 (H1N1)v virus) prepared from virus propagated in the allantoic cavity of embryonated hens' eggs. The manufacturing processes for the D-Pan and Q-Pan H1N1 antigen components were similar to those of their corresponding licensed seasonal influenza vaccines (*Fluari*x™ and *FluLaval*™, respectively). For D-Pan H1N1, the virus was purified by centrifugation and disrupted with sodium deoxycholate^8^, and inactivated by sodium deoxycholate and formaldehyde, then the split virus was further purified by ultrafiltration and sterilized by filtration. For Q-Pan H1N1, the virus was treated with ultraviolet light followed by formaldehyde inactivation, and after purification by centrifugation and disruption with sodium deoxycholate, the split virus was homogenized and sterilized by filtration.

### Sample treatment

Purification of the IgG fraction from the serum samples was performed in order to remove most non-IgG proteins found in serum. For untreated serum samples, the signal of the specific IgG fraction may be obscured by the non-specific protein fraction, as well as the IgM fraction (since the signal measured by SPR biosensors is proportional to a mass/surface unit; 1 RU = 1 pg/mm^2^;^[^^37^^]^). Briefly, serum samples were filtered at 4°C using Vivapure Q devices (Sartorius; #VS-IX01QH24) to remove fibrin and fibrinogen. Next, the IgG fraction was purified from the filtered samples using Melon Gel IgG purification kits (Thermo Scientific; #45206). The purified fractions containing the IgG were subsequently collected, dialyzed in the same buffer as was used for the avidity assessment by Biacore analysis (10mM TRIS 0.75 M NaCl, 5 mM EDTA [pH 7.4] and 0.05% Tween 20), and used for further testing.

### Antigen ligand

The HA external envelope protein, a full-length glycosylated recombinant protein of the H1N1 A/California/07/2009 strain (rHA0) (Protein Sciences, Meriden, USA, #3006) was used to validate binding in the Biacore assay. The protein was produced in insect cells using the baculovirus expression vector system, and purified to obtain >90% purity, under conditions that preserved its biological activity and tertiary structure. Sequence alignment revealed minor differences between the rHA0 amino sequence and the D-Pan or Q-Pan sequences at position 226 (i.e., T in the vaccine antigens, and K in the ligand), and position 240 (i.e., R in the vaccine antigens, and Q in the ligand). At position 146, the rHA0 and D-Pan sequences contained the same amino acid, N, while the Q-Pan sequence contained D, consistent with data from Jacob *et al.*, 2014.^9^

### HI titers

HI titers were assessed as described^12^, both before and after antibody purification. Briefly, 25 µl of serial dilutions of either the serum or the purified IgG fraction were dispensed in 96-well micro-titer plates. Twenty-five µl of A(H1N1)pdm09 influenza antigen (whole inactivated virus) was then added to each dilution, the plates were shaken at room temperature for 60 min, and 50 µl of chicken erythrocyte suspension was added to each well and shaken. Plates were then incubated at room temperature for 75 min. HI titers were determined by the highest dilution at which the hemagglutination was inhibited.

### Antibody avidity assessment by SPR

Kinetic analysis of D-Pan and Q-Pan sera binding to rHA0 was performed on a Biacore T200 instrument (GE Healthcare, Uppsala, Sweden). The rHA0 was immobilized onto a CM5 sensor chip (Biacore, GE Healthcare). Using an amine conjugation kit (Biacore, GE Healthcare), the active flow cell was first activated with a 7-minute injection of equal volumes of 0.05M N-hydroxysuccinimide (NHS) and 0.2 M N-ethyl-N'-diethylaminopropyl carbodiimide (EDC) according to the manufacturer's instructions. The rHA0 protein was then injected at a concentration of 5 µg/ml in 10 mM Na acetate buffer (pH = 5.5) with the 200 RU target level. Last, free non-reacted sulfinimide groups were blocked by a 7-min injection of 1 M ethanolamine hydroxide (pH = 8.5). The first channel was used as a reference surface to measure non-specific binding, for which the same amine chemistry was performed but no ligand was injected, and all sites were blocked with ethanolamine hydroxide. Dulbecco's phosphate saline buffer pH 7.4 (Lonza) supplemented with 0.05% Tween 20 was used as running buffer only for the immobilization. During the sample analysis, the serum samples or the purified IgG fractions were diluted 100-fold in running buffer (10 mM TRIS 0.75 M NaCl, 5 mM EDTA [pH 7.4] and 0.05% Tween 20) and analyzed randomly in a sequence of 10–14 samples, with each sequence comprising a first cycle of conditioning with blank injection, followed by the sample cycles, a control injection with HA-specific control mAb (in order to assess the binding capacity evolution), and, finally, a blank (buffer) injection. Each sequence was analyzed twice. The mouse influenza A(H1N1)pdm09 control IgG mAb (Abd Serotec, #5315–2907) was diluted at 5 µg/ml in the running buffer. The study samples, blank and control were injected for 600 s, during which time association was measured. Dissociation was analyzed for 1000 s at a flow rate of 30 µl/min. The channels were regenerated by two 10 mM glycine-HCl pH 1.7 pulse injections (30 s at 30 µl/min). The analysis was performed at 25°C. The sample compartment temperature was set at 4°C. The k_d_ values were calculated using BIAcore T200 evaluation software (version 1.0), by fitting the $R=R_{0}*e^{-(k_{d}(t-t_{0}))}$equation (where R/R_0_: binding responses at time-points t/t_0_, respectively; t_0:_ stability report point; t: the end of the 1000 s dissociation period). A global fitting on both independent SPR runs was performed on each sample, and the agreement between experimental data and the calculated fits was evaluated. Dissociation data were corrected for non-specific binding by subtraction of the signals obtained for the reference surface and the blank (running buffer).

### Statistical analysis

Descriptive statistical analyses were performed using SAS v9.2 software. Differences in antibody avidity parameters between vaccine groups were estimated by fitting an analysis of variance (ANOVA) model for repeated measures on the log_10_-transformed HI titers (as measured before and after sample purification), end association rates and dissociation rates jointly. Geometric means of both runs were used for the analysis of the latter 2 parameters. Geometric means and GMRs were estimated for each parameter, with 95% confidence intervals for the HI assays, and with 97.5% confidence intervals for the end association and dissociation levels. The latter was performed due to use of a Bonferroni correction to account for the multiplicity, in order to obtain an overall 5% type I error rate on the primary objectives. The correlation between HI titers and end association levels was also estimated through the ANOVA model. As a supplementary analysis, an analysis of covariance (ANCOVA) was fitted on the log_10_ association values in order to estimate the difference between groups after adjustment for the antibody concentration.

## Supplementary Material

Supplementary Figures and Tables

## Trademarks

Arepanrix, Pandemrix, Fluarix and FluLaval are trademarks of the GSK group of companies.

## References

[1] Mereckiene J, Cotter S, Weber JT, Nicoll A, D'Ancona F, Lopalco PL, Johansen K, Wasley AM, Jorgensen P, Lévy-Bruhl D, et al. Influenza A(H1N1)pdm09 vaccination policies and coverage in Europe. Euro Surveill 2012; 17(4):pii=20064; PMID:22297139; http://www.eurosurveillance.org/ViewArticle.aspx?ArticleId=20064 10.2807/ese.17.04.20064-en 22297139

[2] Ahmed SS, Schur PH, MacDonald NE, Steinman L. Narcolepsy, 2009 A(H1N1) pandemic influenza, and pandemic influenza vaccinations: what is known and unknown about the neurological disorder, the role for autoimmunity, and vaccine adjuvants. J Autoimmun 2014; 50:1-11; PMID:24559657; http://dx.doi.org/10.1016/j.jaut.2014.01.033 24559657

[3] European Centre for Disease Prevention and Control Narcolepsy in association with pandemic influenza vaccination - a multi-country European epidemiological investigation). Stockholm: ECDC; 9 2012 Accessed 10-3-2015 http://www.ecdc.europa.eu/en/publications/publications/vaesco%20report%20final%20with%20cover.pdf

[4] Wijnans L, Lecomte C, de Vries C, Weibel D, Sammon C, Hviid A, Svanström H, Mølgaard-Nielsen D, Heijbel H, Dahlström LA, et al. The incidence of narcolepsy in Europe: before, during, and after the influenza A(H1N1)pdm09 pandemic and vaccination campaigns. Vaccine 2013; 31:1246-54; PMID:23246544; http://dx.doi.org/10.1016/j.vaccine.2012.12.015 23246544

[5] Kornum B, Faraco J, Mignot E. Narcolepsy with hypocretin/orexin deficiency, infections and autoimmunity of the brain. Curr Opin Neurobiol 2011; 21:897-903; PMID:21963829; http://dx.doi.org/10.1016/j.conb.2011.09.003 21963829

[6] van der Most R, Van Mechelen M, Destexhe E, Wettendorff M, Hanon E. Narcolepsy and A(H1N1)pdm09 vaccination: Shaping the research on the observed signal. Hum Vaccin Immunother 2014; 10:572-6; PMID:24342916; http://dx.doi.org/10.4161/hv.27412 24342916 PMC4130276

[7] Montplaisir J, Petit D, Quinn MJ, Ouakki M, Deceuninck G, Desautels A, Mignot E, De Wals P. Risk of narcolepsy associated with inactivated adjuvanted (AS03) A/H1N1 (2009) pandemic influenza vaccine in Quebec. PLoS One 2014; 9:e108489; PMID:25264897; http://dx.doi.org/10.1371/journal.pone.0108489 25264897 PMC4180737

[8] European Centre for Disease Prevention and Control Association of receipt of Pandemrix™ and narcolepsy in children and adolescents in the UK (England). Accessed 18-3-2013 http://ecdc.europa.eu/en/activities/sciadvice/_layouts/forms/Review_DispForm.aspx?List=a3216f4c-f040-4f51-9f77-a96046dbfd72&ID=735

[9] Jacob L, Leib R, Ollila HM, Bonvalet M, Adams CM, Mignot E Comparison of Pandemrix and Arepanrix, two pH1N1 AS03-adjuvanted vaccines differentially associated with narcolepsy development. Brain Behav Immun 2014; S0889-1591(14):00519-4 10.1016/j.bbi.2014.11.004 25452148

[10] Vaarala O, Vuorela A, Partinen M, Baumann M, Freitag TL, Meri S, Saavalainen P, Jauhiainen M, Soliymani R, Kirjavainen T, et al. Antigenic differences between AS03 adjuvanted influenza A (H1N1) pandemic vaccines: implications for Pandemrix-associated narcolepsy risk. PLoS One 2014; 9:e114361; PMID:25501681; http://dx.doi.org/10.1371/journal.pone.0114361 25501681 PMC4266499

[11] Ahmed SS, Volkmuth W, Duca J, Corti L, Pallaoro M, Pezzicoli A, Karle A, Rigat F, Rappuoli R, Narasimhan V, et al. Antibodies to influenza nucleoprotein cross-react with human hypocretin receptor 2. Sci Transl Med 2015; 7:294ra105; PMID:26136476; http://dx.doi.org/10.1126/scitranslmed.aab2354 26136476

[12] Launay O, Duval X, Fitoussi S, Jilg W, Kerdpanich A, Montellano M, Schwarz TF, Watanveerade V, Wenzel JJ, Zalcman G, et al. Extended antigen sparing potential of AS03-adjuvanted pandemic H1N1 vaccines in children, and immunological equivalence of two formulations of AS03-adjuvanted H1N1 vaccines: results from two randomised trials. BMC Infect Dis 2013; 13:435; PMID:24041010; http://dx.doi.org/10.1186/1471-2334-13-435 24041010 PMC3848562

[13] Langley JM, Frenette L, Ferguson L, Riff D, Sheldon E, Risi G, Johnson C, Li P, Kenney R, Innis B, et al. Safety and cross-reactive immunogenicity of candidate AS03-adjuvanted prepandemic H5N1 influenza vaccines: a randomized controlled phase 1/2 trial in adults. J Infect Dis 2010; 201:1644-53; PMID:20423222; http://dx.doi.org/10.1086/652701 20423222

[14] Steward MW. The biological significance of antibody affinity. Immunol Today 1981; 2:134-40; PMID:25289455; http://dx.doi.org/10.1016/0167-5699(81)90079-7 25289455

[15] Usinger WR, Lucas AH. Avidity as a determinant of the protective efficacy of human antibodies to pneumococcal capsular polysaccharides. Infect Immun 1999; 67:2366-70; PMID:10225896 10225896 10.1128/iai.67.5.2366-2370.1999 PMC115979

[16] Khurana S, Wu J, Dimitrova M, King LR, Manischewitz J, Graham BS, Ledgerwood JE, Golding H. DNA priming prior to inactivated influenza A(H5N1) vaccination expands the antibody epitope repertoire and increases affinity maturation in a boost-interval-dependent manner in adults. J Infect Dis 2013; 208:413-7; PMID:23633404; http://dx.doi.org/10.1093/infdis/jit178 23633404 PMC3699004

[17] Junker AK, Tilley P. Varicella-zoster virus antibody avidity and IgG-subclass patterns in children with recurrent chickenpox. J Med Virol 1994; 43:119-24; PMID:8083659; http://dx.doi.org/10.1002/jmv.1890430204 8083659

[18] Breukels MA, Jol-van der Zijde E, van Tol MJ, Rijkers GT. Concentration and avidity of anti-*Haemophilus influenzae* type b (Hib) antibodies in serum samples obtained from patients for whom Hib vaccination failed. Clin Infect Dis 2002; 34:191-7; PMID:11740707; http://dx.doi.org/10.1086/338259 11740707

[19] Pedersen GK, Höschler K, Øie Solbak SM, Bredholt G, Pathirana RD, Afsar A, Breakwell L, Nøstbakken JK, Raae AJ, Brokstad KA, et al. Serum IgG titres, but not avidity, correlates with neutralizing antibody response after H5N1 vaccination. Vaccine 2014; 32:4550-7; PMID:24950357; http://dx.doi.org/10.1016/j.vaccine.2014.06.009 24950357

[20] Bachmann MF, Kalinke U, Althage A, Freer G, Burkhart C, Roost H, Aguet M, Hengartner H, Zinkernagel RM. The role of antibody concentration and avidity in antiviral protection. Science 1997; 276:2024-7; PMID:9197261; http://dx.doi.org/10.1126/science.276.5321.2024 9197261

[21] Olotu A, Clement F, Jongert E, Vekemans J, Njuguna P, Ndungu FM, Marsh K, Leroux-Roels G, Bejon P. Avidity of anti-circumsporozoite antibodies following vaccination with RTS,S/AS01_E_ in young children. PLoS One 2014; 9:e115126; PMID:25506706; http://dx.doi.org/10.1371/journal.pone.0115126 25506706 PMC4266636

[22] Monsalvo AC, Batalle JP, Lopez MF, Krause JC, Klemenc J, Hernandez JZ, Maskin B, Bugna J, Rubinstein C, Aguilar L, et al. Severe pandemic 2009 H1N1 influenza disease due to pathogenic immune complexes. Nat Med 2011; 17:195-9; PMID:21131958; http://dx.doi.org/10.1038/nm.2262 21131958 PMC3034774

[23] To KK, Zhang AJ, Hung IF, Xu T, Ip WC, Wong RT, Ng JC, Chan JF, Chan KH, Yuen KY. High titer and avidity of nonneutralizing antibodies against influenza vaccine antigen are associated with severe influenza. Clin Vaccine Immunol 2012; 19:1012-8; PMID:22573737; http://dx.doi.org/10.1128/CVI.00081-12 22573737 PMC3393364

[24] Khurana S, Verma N, Yewdell JW, Hilbert AK, Castellino F, Lattanzi M, Del Giudice G, Rappuoli R, Golding H. MF59 adjuvant enhances diversity and affinity of antibody-mediated immune response to pandemic influenza vaccines. Sci Transl Med 2011; 3:85ra48; PMID:21632986; http://dx.doi.org/10.1126/scitranslmed.3002336 21632986 PMC3501657

[25] Khurana S, Verma N, Talaat KR, Karron RA, Golding H. Immune response following H1N1pdm09 vaccination: differences in antibody repertoire and avidity in young adults and elderly populations stratified by age and gender. J Infect Dis 2012; 205:610-20; PMID:22207649; http://dx.doi.org/10.1093/infdis/jir791 22207649

[26] Khurana S, Chearwae W, Castellino F, Manischewitz J, King LR, Honorkiewicz A, Rock MT, Edwards KM, Del Giudice G, Rappuoli R, et al. Vaccines with MF59 adjuvant expand the antibody repertoire to target protective sites of pandemic avian H5N1 influenza virus. Sci Transl Med 2010; 2:15ra5; PMID:20371470; http://dx.doi.org/10.1126/scitranslmed.3000624 20371470

[27] Lynch HE, Stewart SM, Kepler TB, Sempowski GD, Alam SM. Surface plasmon resonance measurements of plasma antibody avidity during primary and secondary responses to anthrax protective antigen. J Immunol Methods 2014; 404:1-12; PMID:24316020; http://dx.doi.org/10.1016/j.jim.2013.11.026 24316020 PMC4104170

[28] Dauner JG, Pan Y, Hildesheim A, Kemp TJ, Porras C, Pinto LA. Development and application of a GuHCl-modified ELISA to measure the avidity of anti-HPV L1 VLP antibodies in vaccinated individuals. Mol Cell Probes 2012; 26:73-80; PMID:22285687; http://dx.doi.org/10.1016/j.mcp.2012.01.002 22285687 PMC3319198

[29] Hearty S, Conroy PJ, Ayyar BV, Byrne B, O'Kennedy R. Surface plasmon resonance for vaccine design and efficacy studies: recent applications and future trends. Expert Rev Vaccines 2010; 9:645-64; PMID:20518719; http://dx.doi.org/10.1586/erv.10.52 20518719

[30] Harris T, Wong K, Stanford L, Fediurek J, Crowcroft N, Deeks S. Did narcolepsy occur following administration of AS03-adjuvanted A(H1N1) pandemic vaccine in Ontario, Canada? A review of post-marketing safety surveillance data. Euro Surveill 2014; 19(36):pii=20900; PMID:25232921; http://www.eurosurveillance.org/ViewArticle.aspx?ArticleId=20900 10.2807/1560-7917.es2014.19.36.20900 25232921

[31] Stiehm ER, Fudenberg HH. Serum levels of immune globulins in health and disease: a survey. Pediatrics 1966; 37:715-27; PMID:4956666 4956666

[32] Katsamba PS, Navratilova I, Calderon-Cacia M, Fan L, Thornton K, Zhu M, Vanden Bos T, Forte C, Friend D, Laird-Offringa I, et al. Kinetic analysis of a high-affinity antibody/antigen interaction performed by multiple Biacore users. Anal Biochem 2006; 352:208-21; PMID:16564019; http://dx.doi.org/10.1016/j.ab.2006.01.034 16564019

[33] García-Ojeda PA, Hardy S, Kozlowski S, Stein KE, Feavers IM. Surface plasmon resonance analysis of antipolysaccharide antibody specificity: responses to meningococcal group C conjugate vaccines and bacteria. Infect Immun 2004; 72:3451-60; PMID:Can't; http://dx.doi.org/10.1128/IAI.72.6.3451-3460.2004 15155652 PMC415682

[34] Gibbs E, Karim ME, Oger J. Antibody dissociation rates are predictive of neutralizing antibody (NAb) course: A comparison of interferon beta-1b-treated Multiple Sclerosis (MS) patients with transient versus sustained NAbs. Clin Immunol 2014; 157:91-101; PMID:25543089; http://dx.doi.org/10.1016/j.clim.2014.12.005 25543089

[35] European Medicines Agency European Public Assessment Report (EPAR) for Pandemrix, EMA/691037/2013. Annex I: Summary of product characteristics Accessed 13-3-2013 http://www.ema.europa.eu/ema/index.jsp?curl=pages/medicines/human/medicines/000832/human_med_000965.jsp&mid=WC0b01ac058001d124

[36] Leroux-Roels I, Borkowski A, Vanwolleghem T, Drame M, Clement F, Hons E, Devaster JM, Leroux-Roels G. Antigen sparing and cross-reactive immunity with an adjuvanted rH5N1 prototype pandemic influenza vaccine: a randomised controlled trial. Lancet 2007; 370:580-9; PMID:17707753; http://dx.doi.org/10.1016/S0140-6736(07)61297-5 17707753

[37] Stenberg E, Persson B, Roos H, Urbaniczky C Quantitative determination of surface concentration of protein with surface plasmon resonance using radiolabeled proteins. J Colloid Interface Sci 1991; 143:513-26; http://dx.doi.org/10.1016/0021-9797(91)90284-F

