# Supplementary material for: Evaluation of potential immunogenicity differences between Pandemrix™ and Arepanrix™
Source: Hum Vaccin Immunother. 2016 Apr 22;12(9):2289–98. doi: 10.1080/21645515.2016.1168954 (PMC5027709; doi:10.1080/21645515.2016.1168954)
Supplement: Supplementary Figures and Tables [file khvi-12-09-1168954-s001.zip › Supplementary Table 1.docx]

**Supplementary Table 1. HI titers and association, dissociation and k_d_ values for the D-Pan and Q-Pan sera.**

| **Treatment** | **Sample no.** | **HI titer** | |  | **Association (RU)** | |  | **Dissociation (%)** | | **k_d (s_^-1^_)_** |
| --- | --- | --- | --- | --- | --- | --- | --- | --- | --- | --- |
|  |  | **before purification** | **purified IgG fraction** |  | **run1** | **run2** |  | **run1** | **run2** |  |
| D-Pan | 251 | 905 | 320 |  | 13.3 | 12.3 |  | 95.7 | 98.3 | 3.63 × 10^-5^ |
|  | 254 | 640 | 320 |  | 14.3 | 13.2 |  | 96.2 | 95.7 | 3.63 × 10^-5^ |
|  | 261 | 1810 | 640 |  | 26.1 | 22.6 |  | 91.3 | 90.3 | 9.16 × 10^-5^ |
|  | 263 | 905 | 640 |  | 18.8 | 16 |  | 95.9 | 94 | 4.71 × 10^-5^ |
|  | 265 | 640 | 320 |  | 14.5 | 13.5 |  | 101.2 | 102.7 | 1.94 × 10^-8^ |
|  | 276 | 1280 | 453 |  | 13.9 | 12.4 |  | 93.9 | 96.7 | 5.74 × 10^-5^ |
|  | 281 | 2560 | 905 |  | 26.9 | 22.7 |  | 89.2 | 88.3 | 1.08 × 10^-4^ |
|  | 283 | 320 | 160 |  | 9.9 | 8.8 |  | 100 | 96.2 | 4.08 × 10^-5^ |
|  | 285 | 453 | 160 |  | 9.7 | 9 |  | 93.9 | 96.4 | 6.85 × 10^-5^ |
|  | 289 | 640 | 453 |  | 19.3 | 19.1 |  | 97.7 | 99.2 | 1.99 × 10^-5^ |
|  | 292 | 1280 | 640 |  | 22.4 | 20.3 |  | 93.8 | 93.6 | 5.75 × 10^-5^ |
|  | 294 | 1810 | 1280 |  | 29.4 | 23.5 |  | 94.3 | 94.3 | 5.50 × 10^-5^ |
|  | 299 | 905 | 453 |  | 19.6 | 18.5 |  | 95 | 95.1 | 5.11 × 10^-5^ |
|  | 305 | 1810 | 640 |  | 34.5 | 28.8 |  | 91.5 | 91.1 | 8.24 × 10^-5^ |
|  | 309 | 905 | 453 |  | 17.6 | 14.9 |  | 91.9 | 93.2 | 7.23 × 10^-5^ |
|  | 312 | 640 | 453 |  | 15.2 | 14.2 |  | 94.6 | 95.1 | 4.95 × 10^-5^ |
|  | 314 | 905 | 453 |  | 15.9 | 13.8 |  | 89.9 | 90.9 | 1.08 × 10^-4^ |
|  | 318 | 905 | 640 |  | 14.3 | 12.9 |  | 96.4 | 97.1 | 3.10 × 10^-5^ |
|  | 322 | 320 | 226 |  | 8.8 | 8.1 |  | 100 | 105.9 | 4.11 × 10^-8^ |
|  | 324* | 1810 | 905 |  | 7.9 | 7.6 |  | 546.7 | 364.7 | 2.05 × 10^-7^ |
|  | 329 | 2560 | 1280 |  | 22.4 | 18 |  | 91.5 | 91.6 | 7.90 × 10^-5^ |
|  | 334 | 905 | 453 |  | 15.1 | 13.1 |  | 98.9 | 95.7 | 3.54 × 10^-5^ |
|  | 339 | 1810 | 640 |  | 16.6 | 13.7 |  | 95.1 | 94.6 | 5.35 × 10^-5^ |
|  | 341 | 453 | 160 |  | 10.4 | 10 |  | 94.9 | 97.3 | 3.87 × 10^-5^ |
|  | 343 | 1810 | 905 |  | 18.4 | 14.6 |  | 98.4 | 101.2 | 1.07 × 10^-5^ |
|  | 346 | 226 | 160 |  | 8.7 | 8.4 |  | 81.8 | 83.3 | 1.46 × 10^-4^ |
|  | 348 | 640 | 320 |  | 12.5 | 10.8 |  | 91.5 | 91.7 | 8.04 × 10^-5^ |
|  | 351 | 2560 | 905 |  | 24.2 | 20.7 |  | 91.2 | 90.5 | 8.57 × 10^-5^ |
| Q-Pan | 253 | 1280 | 640 |  | 17.8 | 15.8 |  | 89 | 88.7 | 1.08 × 10^-4^ |
|  | 256* | 80 | 40 |  | 6.8 | 7.3 |  | 128.6 | 133.3 | 7.84 × 10^-8^ |
|  | 259 | 640 | 453 |  | 10.7 | 9.6 |  | 95.2 | 97.2 | 3.57 × 10^-5^ |
|  | 262* | 453 | 160 |  | 6.5 | 6.3 |  | 125 | . | 4.82 × 10^-8^ |
|  | 273 | 2560 | 1280 |  | 27.2 | 23 |  | 95.7 | 94.6 | 4.71 × 10^-5^ |
|  | 277 | 2560 | 905 |  | 17.1 | 14.3 |  | 101.8 | 101.3 | 1.46 × 10^-7^ |
| Q-Pan | 280 | 1280 | 640 |  | 19.9 | 16.5 |  | 98.6 | 97.1 | 1.41 × 10^-5^ |
|  | 282 | 320 | 453 |  | 8.3 | 7.9 |  | 79.2 | 84.2 | 1.67 × 10^-4^ |
|  | 287 | 320 | 160 |  | 11.9 | 11.4 |  | 85.7 | 88.2 | 1.27 × 10^-4^ |
|  | 288 | 160 | 57 |  | 9.2 | 8.8 |  | 94.3 | 96 | 7.90 × 10^-5^ |
|  | 293 | 640 | 320 |  | 9.3 | 8.8 |  | 93.1 | 96 | 5.32 × 10^-5^ |
|  | 295 | 640 | 226 |  | 8.8 | 7.9 |  | 95.5 | 94.7 | 4.82 × 10^-5^ |
|  | 300 | 1810 | 453 |  | 16.1 | 14.7 |  | 95.8 | 95.4 | 4.48 × 10^-5^ |
|  | 303 | 1280 | 640 |  | 18.5 | 16.6 |  | 95.9 | 94.3 | 5.36 × 10^-5^ |
|  | 308 | 905 | 453 |  | 10.3 | 9.5 |  | 94.9 | 96.9 | 4.55 × 10^-5^ |
|  | 310 | 1810 | 1280 |  | 22.9 | 18.6 |  | 96.4 | 95.9 | 3.64 × 10^-5^ |
|  | 313 | 160 | 80 |  | 8.4 | 7.7 |  | 95 | 100 | 5.95 × 10^-5^ |
|  | 323 | 640 | 320 |  | 10.5 | 9.6 |  | 109.8 | 106.1 | 1.10 × 10^-8^ |
|  | 325 | 640 | 453 |  | 12.5 | 11.9 |  | 101.5 | 100 | 2.07 × 10^-8^ |
|  | 328 | 1810 | 905 |  | 14.8 | 12.4 |  | 92.7 | 90.6 | 7.31 × 10^-5^ |
|  | 331 | 1810 | 905 |  | 14.9 | 13.8 |  | 92.9 | 92 | 6.69 × 10^-5^ |
|  | 333 | 1810 | 905 |  | 20 | 18.3 |  | 96.3 | 96.6 | 3.65 × 10^-5^ |
|  | 337 | 1280 | 640 |  | 12.6 | 10.8 |  | 96.7 | 93.8 | 3.90 × 10^-5^ |
|  | 338 | 320 | 226 |  | 8.8 | 8.2 |  | 100 | 110.5 | 1.91 × 10^-8^ |
|  | 342 | 905 | 453 |  | 9.2 | 8.2 |  | 100 | 95.5 | 2.90 × 10^-6^ |
|  | 344 | 640 | 226 |  | 9 | 8.4 |  | 107.7 | 109.1 | 1.88 × 10^-8^ |
|  | 349 | 80 | 57 |  | 9.9 | 9.4 |  | 90.5 | 93.3 | 8.73 × 10^-5^ |
|  | 352 | 3620 | 1280 |  | 28.9 | 24 |  | 88.8 | 86 | 1.20 × 10^-4^ |

*, sample not used for statistical analyses: #324 due to a non-specific signal impeding the SPR analysis, and #256 and #262 due to yielding association rates of less than 1 RU above the level of the blank. RU, resonance units.
