# Supplementary material for: Evaluation of potential immunogenicity differences between Pandemrix™ and Arepanrix™
Source: Hum Vaccin Immunother. 2016 Apr 22;12(9):2289–98. doi: 10.1080/21645515.2016.1168954 (PMC5027709; doi:10.1080/21645515.2016.1168954)

Suppl. Figure 1: Non-specific binding. Levels of non-specific binding of the serum matrix were determined for purified serum samples and the control (an A(H1N1)pdm09 HA-specific mouse monoclonal antibody). Serum samples were obtained at 3 weeks post vaccination from children who received either the D-Pan or Q-Pan vaccine, and were then purified to reduce the fractions of non-specific protein and IgM. The results shown were obtained for samples containing the purified IgG fraction. Symbols represent the averages of 2 analysis runs per sample. Presented values were corrected for the signal of the running buffer injection. RU, resonance units.

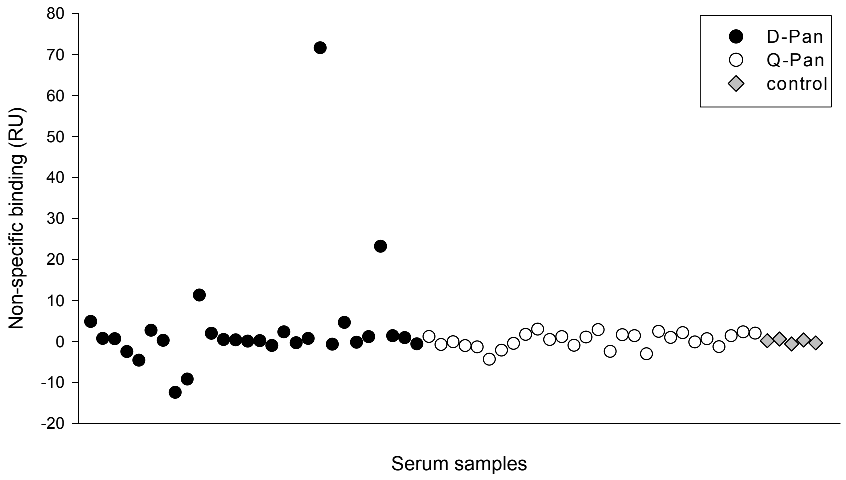

Supplement: Supplementary Figures and Tables [file khvi-12-09-1168954-s001.zip › Supplement Figure 1.pdf]
