# Supplementary material for: Evaluation of potential immunogenicity differences between Pandemrix™ and Arepanrix™
Source: Hum Vaccin Immunother. 2016 Apr 22;12(9):2289–98. doi: 10.1080/21645515.2016.1168954 (PMC5027709; doi:10.1080/21645515.2016.1168954)

Suppl. Figure 2. Distribution of HI titers before and after sample purification. Serum samples were obtained 3 weeks after vaccination from children who received either the D-Pan or Q-Pan vaccine. Distributions of the log-transformed hemagluttination inhibition (HI) titers were determined for the serum samples before purification (**A**) and for the purified IgG fraction (**B**). Samples were purified to reduce the fractions of IgM and non-specific protein. Box-and-whiskers plots represent the medians and interquartile range (boxes), minimum and maximum values (whiskers). Diamonds and open circles represent means and outliers, respectively. Values above the 75th percentile + 1.5 IQR, or below the 25th percentile – 1.5 IQR, were considered outliers.

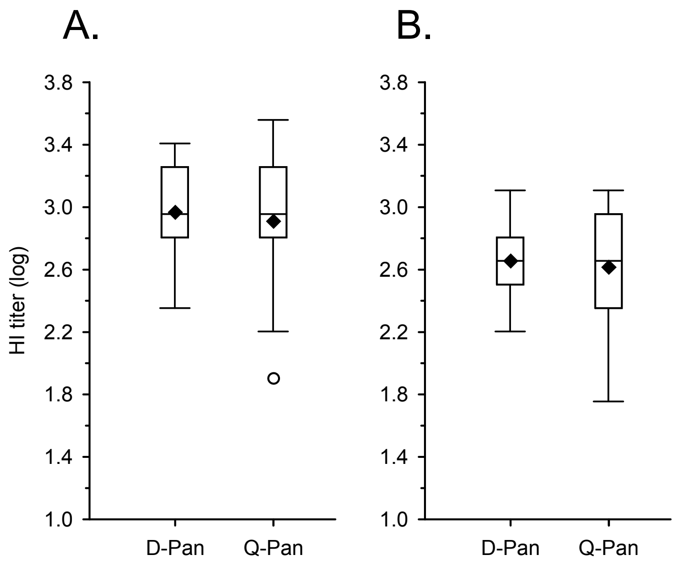

Supplement: Supplementary Figures and Tables [file khvi-12-09-1168954-s001.zip › Supplement Figure 2.pdf]
